# Calcific Uremic Arteriolopathy in End-Stage Renal Disease: A Rare Life-Threatening Condition

**DOI:** 10.7759/cureus.48002

**Published:** 2023-10-30

**Authors:** Tetyana Okan, Kulbir Ghuman, Faiza Chaudhary, Kaushik Doshi, Usman Shoaib

**Affiliations:** 1 Department of Internal Medicine, Jamaica Hospital Medical Center, New York, USA

**Keywords:** calciphylaxis, calcific uremic arteriolopathy, end-stage renal disease (esrd), dialysis, sodium thiosulfate, wound care, mortality

## Abstract

Calcific uremic arteriolopathy, or calciphylaxis, is a highly morbid, life-threatening syndrome of microvascular calcification leading to progressive skin necrosis. It affects 1-4% of the population with end-stage renal disease (ESRD) and is rarely seen in other conditions. The one-year mortality rate is 80% for patients with ulcerations. There is no evidence-based treatment, and the response to therapy is poor; thus, it creates a serious therapeutic challenge for clinicians. Our aim is to discuss a diagnostic approach and management of calciphylaxis and to raise awareness about this condition among the medical community. We are describing a case of calciphylaxis in a 68-year-old female with a past medical history of ESRD on hemodialysis, who presented with altered mental status and painful ulcers on her feet and thighs. The patient was admitted for acute encephalopathy due to sepsis secondary to a urinary tract infection (UTI) versus permacath-related bacteremia versus wound infection of pressure ulcers on the lower extremities. Broad-spectrum antibiotics were started. Computed tomography (CT) of bilateral thighs with contrast showed extensive arterial calcifications, characteristic of calciphylaxis. Nephrology recommended sodium thiosulfate with each hemodialysis session. Vitamin D and iron were discontinued. Despite therapy, the wound significantly progressed within the next eight weeks, leading to mortality due to sepsis. In conclusion, calcific uremic arteriolopathy is a challenging disease with a poor prognosis. Early diagnosis is crucial, so aggressive therapy can be started immediately. A multidisciplinary approach may improve survival in cases of calciphylaxis. More studies are needed to improve the diagnostic approach and medical management of the disease.

## Introduction

Calcific uremic arteriolopathy, or calciphylaxis, is a highly morbid syndrome of extensive microvascular calcification leading to progressive skin necrosis. It affects 1-4% of the population with end-stage renal disease (ESRD) and is rarely seen in other conditions [1]. However, the incidence of calciphylaxis has increased during the last decade, possibly due to the more widespread use of parenteral vitamin D and iron dextran [2]. Calciphylaxis has been reported in individuals ranging in age from 6 months to 83 years. It is more prevalent in the white population, with a female-to-male ratio of 3:1 [2]. The pathogenesis of calciphylaxis is poorly understood. It involves arteriolar mural calcification, intimal hyperplasia, and endovascular fibrosis, which cause a reduction in arterial blood flow and subsequently skin lesions [3,4]. Additional vascular damage, including local trauma, hypotension, or thrombus formation, leads to the development of ischemic infarcts and ulcerations [4]. Hyperparathyroidism, chronic inflammation, and deficiencies in inhibitors of vascular calcification may also play a role [3]. This pathology is associated with high morbidity due to severe pain, non-healing wounds, and frequent hospitalizations. Approximately 50% of patients are bedridden or wheelchair-bound, and more than 70% require hospitalization for severe ulcers [3]. There is no evidence-based treatment for calcific uremic arteriolopathy. The response to therapy and prognosis of the disease are poor, creating a serious therapeutic challenge for clinicians [2].

## Case presentation

**Figure 1:**
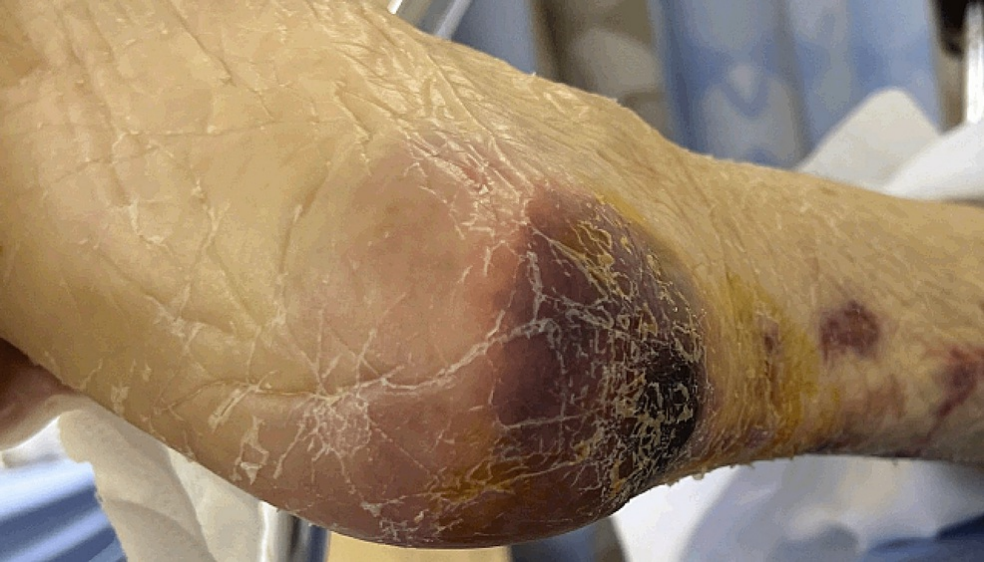
Painful tissue injury on the right heel.

A 68-year-old female with a past medical history of essential hypertension, atrial fibrillation, type 2 diabetes mellitus, and ESRD on hemodialysis presented with altered mental status from a nursing home. The patient was afebrile but hypotensive, requiring fluids and pressor support. She complained of weakness and painful ulcers on both her feet and thighs. The ulcers had been present since her prior hospital admission for septic shock due to a Candida urinary tract infection (UTI) one month ago and have progressively increased in size and become tender. The patient denied fever, chills, chest pain, shortness of breath, headache, dizziness, and urinary symptoms. Her past medical history was significant for a recent embolic stroke. Past surgical history included the placement of an interventional radiology tunneled central venous dialysis catheter two months ago. Prior to admission, her medications included amlodipine, carvedilol, apixaban, sitagliptin/metformin, atorvastatin, vitamin D, calcium carbonate, epoetin alfa, and sevelamer. At baseline, she was bedbound and dependent for activities of daily living. The patient was alert and oriented to person and place only. On a neurological exam, weakness was noted in the left upper extremity and bilateral lower extremities, graded at 4/5. On physical examination of the lower extremities, there was a 12 x 5 cm extremely tender, irregularly shaped necrotic wound on the left lateral thigh extending posteriorly. Additionally, a similar wound was noted on the right lower medial thigh. The right heel had a dark red tissue injury measuring 6 x 8 cm (Figure 1).

**Figure 2:**
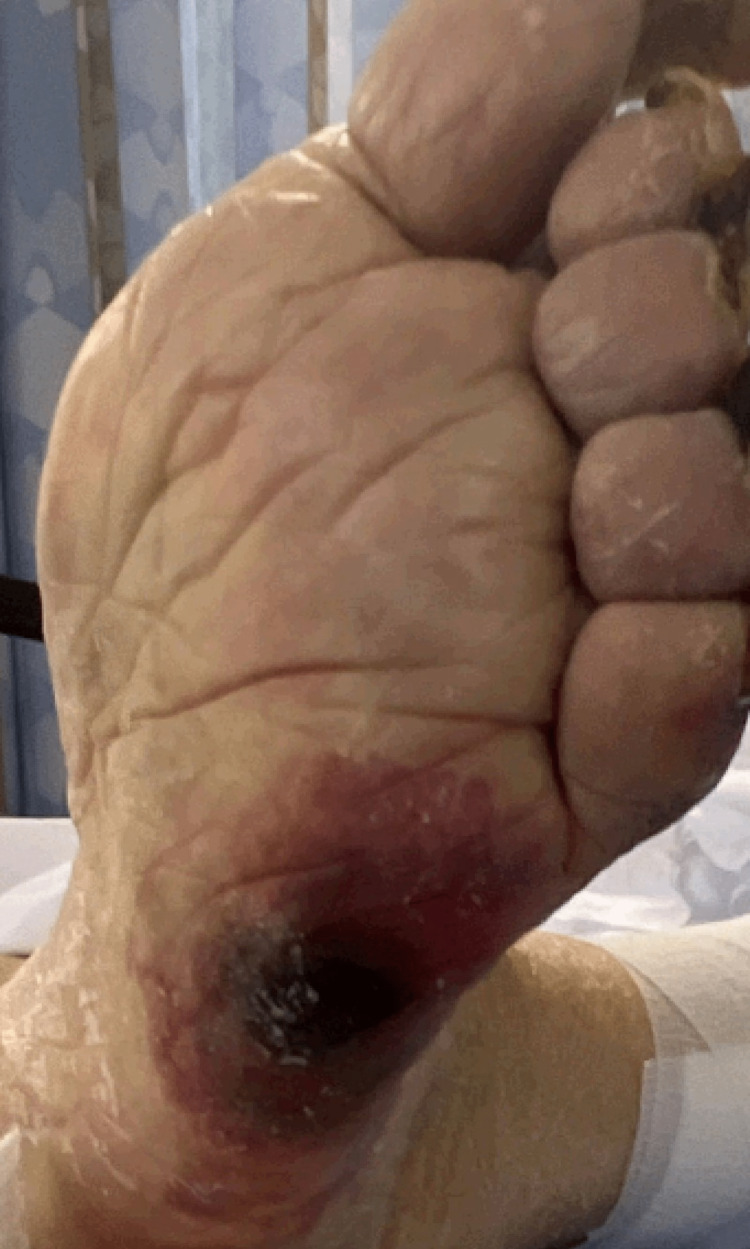
Painful tissue injury of the lateral aspect of the left foot.

The lateral aspect of the left foot had a similar painful tissue injury, 6 x 6 cm (Figure 2).

Initial labs were notable for neutrophilic leukocytosis of 24 x 10^9/L and chronic normocytic anemia. Initial urinalysis suggested a UTI, prompting the start of broad-spectrum antibiotics: zosyn and vancomycin. The patient was admitted for further evaluation of acute encephalopathy, likely due to severe sepsis secondary to a UTI, permacath-related bacteremia, or wound infection of pressure ulcers on the lower extremities. Subsequent blood tests showed elevated creatinine at 4.8 mg/dL, GFR < 15 ml/min, procalcitonin at 52.6 ng/mL, CRP at 24 mg/dL, hypokalemia at 3.3 mmol/L, and mild hypocalcemia at 6.6 mg/dL (corrected calcium 8.2 mg/dL), which were resolved with supplements. During the hospital course, blood cultures grew Clostridium perfringens and Gram-positive rods, possibly secondary to lower extremity pressure ulcers. Urine culture returned negative. An electrocardiogram showed atrial fibrillation and QT prolongation, possibly due to hypocalcemia. Chest X-ray did not reveal any evidence of an infectious process or fluid overload. Wound care was consulted and dressing initiated. Oxycodone with acetaminophen was administered for pain control. Neurology also consulted the patient. Computed tomography (CT) of the head was negative for intracranial hemorrhage. Magnetic resonance imaging of the brain showed old bilateral subacute cerebrovascular accidents. Infectious disease was consulted for a potential tunneled central venous catheter-related bloodstream infection. The tunneled catheter was replaced. Repeat blood culture returned negative.

**Figure 3:**
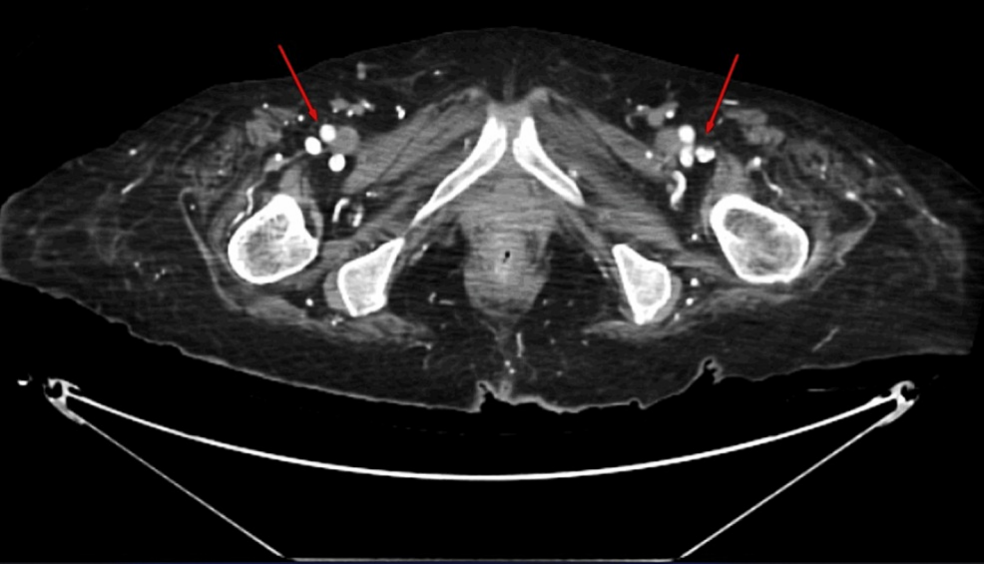
Computed tomography of the thigh with contrast showed extensive arterial calcifications.

**Figure 4:**
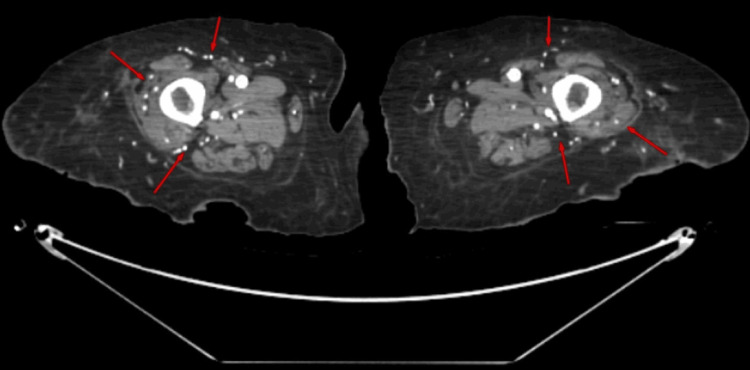
Computed tomography of the lower extremities showed extensive arterial calcifications.

The surgical team concluded that there was low clinical suspicion for the ulcer being the source of bacteremia. The patient's wound appeared suspicious for calciphylaxis in the setting of ESRD on hemodialysis. A CT scan of the bilateral thighs and lower extremities with contrast showed extensive arterial calcifications in both thighs and lower extremities (Figures 3, 4), as well as ruled out gas gangrene or abscess.

Nephrology followed up with possible calciphylaxis. The patient was started on sodium thiosulfate, as the only available treatment option for calciphylaxis. She received 25 g intravenous injections three times per week with hemodialysis. Additionally, 1 unit of blood was transfused and epoetin alfa was given due to a low hemoglobin level of 6.9 g/dl. The phosphorus level was elevated (5.5 g/dL), which was corrected with sevelamer. Nephrocaps (renal vitamins) were recommended daily. Per the dermatology team, a biopsy was not necessary to confirm the diagnosis of calciphylaxis as radiological evidence was already present on the obtained CT scan. It was recommended to avoid vitamin D, warfarin, or iron supplements, as they could worsen the condition. The patient's wound on the left thigh increased during hospitalization from 12 cm up to 14 cm, increasing the risk for mortality due to sepsis for the next one year. Taking into consideration the rapid progression of the disease, palliative care consultation was provided.

The patient's mental status and general condition improved, and she was discharged to a subacute rehabilitation facility. Cefazolin was prescribed after each hemodialysis session for a 14-day period.

**Figure 5:**
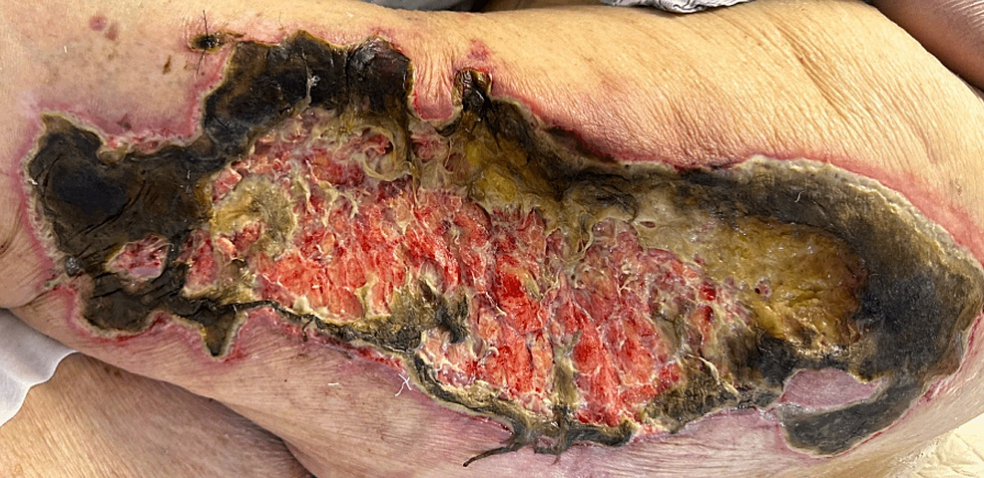
Deep wound of the left lateral thigh with exposure of subcutaneous fat and muscles.

Eight weeks later, the patient's condition significantly deteriorated. She was rehospitalized for septic shock and appeared cachectic, unresponsive to verbal, tactile, or painful stimuli. The wounds had increased in number, size, and depth. On physical examination, the wound on the left lateral thigh had deepened significantly, exposing subcutaneous fat and muscle (Figure 5).

**Figure 6:**
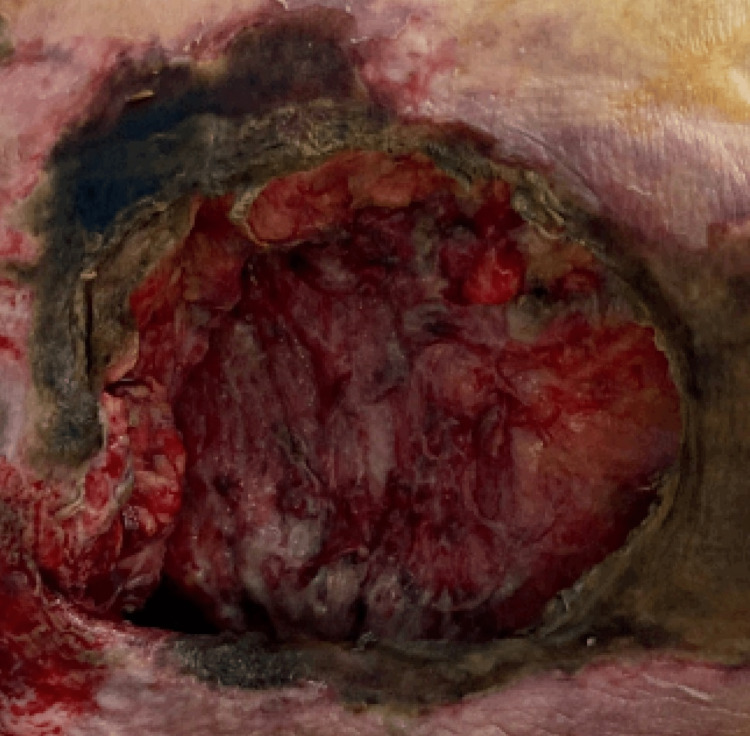
Necrotic wound of the sacrum.

In addition, a large necrotic wound of the sacrum was noticed during the physical examination (Figure 6). 

The palliative care team discussed the goals of care with the family, who chose the DNR/DNI (do not resuscitate/do not intubate) code with comfort measures only. The patient expired within a week.

## Discussion

Calcific uremic arteriolopathy is a rare, poorly understood, life-threatening syndrome of vascular calcification and skin necrosis. It develops more frequently in patients with chronic renal failure. Rarely, it may occur in patients with other conditions such as diabetes mellitus, hypercalcemia, hyperphosphatemia, secondary hyperparathyroidism, obesity, hypercoagulable states, liver disease, metastatic cancer, inflammatory, and autoimmune processes [2,3]. Additional risk factors that promote vascular calcification include corticosteroid use, aluminum exposure, iron dextran infusions, and warfarin therapy [2]. Interestingly, calciphylaxis may also occur in the setting of normal or minimally elevated calcium and phosphate levels. The exact role of parathyroid hormone (PTH) is uncertain, as the disease may occur after total parathyroidectomy in the absence of PTH.

The initial skin manifestations may include induration, livedo or purpura, painful subcutaneous nodules or plaques, eventually leading to nonhealing ulcers or cutaneous necrosis [3]. Usually, the lesions occur in areas where body fat is most abundant, such as the thighs, the buttocks, and the lower part of the abdomen. Fatty areas are at higher risk for thrombosis due to decreased blood flow, increased potential for vascular kinking, and adipocyte involvement in the pathogenesis of calciphylaxis [2].

Diagnosis is based on physical examination findings, which include classic painful lesions covered by a black eschar. There are no specific laboratory findings. In some patients, PTH levels, phosphorus, and calcium may be increased. CT angiography of the extremities can be used to rule out other conditions. Bone scintigraphy may be used as a noninvasive diagnostic tool because the bone matrix protein osteopontin has recently been demonstrated in calciphylaxis lesions [1]. A biopsy is not required for diagnosis, as there is a high rate of false-negative results. Only 45% of obtained histological samples show calcium in the vessel wall or any evidence of calciphylaxis [2]. However, a skin biopsy is indicated if the patient presents with atypical or early lesions, or with classic calciphylaxis morphology but without chronic kidney disease [5]. Histological findings include a mixed inflammatory infiltrate, subintimal fibrosis, dermo-hypodermal and arteriolar calcification, and adipose tissue necrosis [4].

Differential diagnosis of calcific uremic arteriolopathy includes dermatological, vascular, rheumatological, and infectious diseases, such as lower extremity pressure ulcers, cellulitis, atherosclerotic peripheral vascular disease, venous stasis ulcers, erythema nodosum, hypersensitivity vasculitis, pyoderma gangrenosum, purpura fulminans, cryoglobulinemia, coumarin necrosis, cholesterol embolization, Martorell's ulcer, nephrogenic systemic fibrosis, oxalosis, necrotizing vasculitis, and subacute bacterial endocarditis [3,4].

The treatment of calciphylaxis is still experimental and requires a multidisciplinary approach involving wound care, pain management, nephrology, dermatology, surgery, and palliative care. It begins with wound care and pain management. Suspected wound infection necessitates antimicrobial therapy and surgical debridement. Pain can be difficult to manage; thus, multimodal pain control with analgesics with different mechanisms of action should be used. It is important to avoid iron dextran infusions and warfarin therapy, discontinue vitamin D and calcium supplementation, correct calcium and phosphate levels, and use non-calcium-containing phosphate binders (sevelamer and lanthanum carbonate) instead of calcium-containing phosphate binders [4]. Bisphosphonates may be helpful in some cases of calciphylaxis. Calcimimetics such as cinacalcet hydrochloride, as well as parathyroidectomy, may be beneficial in cases of hyperparathyroidism [1].

Significant improvement of calciphylaxis has been reported with the use of intravenous sodium thiosulfate in both uremic and nonuremic cases, although the therapy is not approved [1]. Sodium thiosulfate is known as an antidote for cyanide intoxication. However, it also has antioxidative and vasodilation properties and prevents crystal formation and vascular calcification, thus being effective for patients with calciphylaxis [4]. Sodium thiosulfate is not dependent on plasma calcium levels. Its initial dose is 25 g, given intravenously three times per week after hemodialysis. The dose for patients weighing less than 60 kg is reduced to 12.5 g. If patients do not tolerate intravenous therapy well, intralesional therapy can be administered. The optimal duration of treatment is not known, but for most patients, it lasts for three months. Possible side effects of sodium thiosulfate include high anion gap metabolic acidosis, nausea and vomiting, volume overload, hypocalcemia, and QT interval prolongation [4].

Once calciphylaxis has been diagnosed, the prognosis is generally poor. It is associated with high morbidity due to severe pain, non-healing wounds, and frequent hospitalizations. Approximately 50% of patients are bedridden or wheelchair-bound, and more than 70% require hospitalization for severe ulcers [3]. Skin lesions due to calciphylaxis become ulcers and wounds in the late stages, thus, the most frequent cause of death is sepsis. The estimated one-year mortality rate for all patients with calciphylaxis is up to 45.8%. Patients with ulceration and ESRD demonstrate 80% mortality within one year. The mortality rate is higher in patients with proximal disease than in those with only distal or acral lesions [6]. 

## Conclusions

Calcific uremic arteriolopathy is a challenging diagnosis with poor prognosis and high mortality. It may occur not only in patients with ESRD but also in patients with a variety of other conditions. It is important to be aware of this pathology and avoid medications and supplements that worsen the disease. Early diagnosis is crucial, so aggressive therapy can be started immediately. A multidisciplinary approach involving wound care, aggressive medical management, surgical debridement, and possible parathyroidectomy may improve quality of life and survival in calciphylaxis. More studies are needed to improve the diagnostic approach and medical management of the disease.
